# Individual and Community Social Determinants of Health Associated With Diabetes Management in a Mexican American Population

**DOI:** 10.3389/fpubh.2020.633340

**Published:** 2021-02-03

**Authors:** Kehe Zhang, Belinda Reininger, Miryoung Lee, Qian Xiao, Cici Bauer

**Affiliations:** ^1^Department of Biostatistics and Data Science, School of Public Health, The University of Texas Health Science Center at Houston, Houston, TX, United States; ^2^Department of Health Promotion and Behavior Sciences, School of Public Health, The University of Texas Health Science Center at Houston, Brownsville Regional Campus, Brownsville, TX, United States; ^3^Department of Epidemiology, Human Genetics and Environmental Science, School of Public Health, The University of Texas Health Science Center at Houston, Brownsville, TX, United States; ^4^Department of Epidemiology, Human Genetics and Environmental Science, School of Public Health, The University of Texas Health Science Center at Houston, Houston, TX, United States

**Keywords:** diabetes, social determinants of health, Mexican Americans, chronic disease management, multilevel modeling, Bayesian, spatial

## Abstract

**Background:** Diabetes is a major health burden in Mexican American populations, especially among those in the Lower Rio Grande Valley (LRGV) in the border region of Texas. Understanding the roles that social determinants of health (SDOH) play in diabetes management programs, both at the individual and community level, may inform future intervention strategies.

**Methods:** This study performed a secondary data analysis on 1,568 individuals who participated in *Salud y Vida* (SyV), a local diabetes and chronic disease management program, between October 2013 and September 2018 recruited from a local clinic. The primary outcome was the reduction of hemoglobin A1C (HbA1C) at the last follow-up visit compared to the baseline. In addition to age, gender, insurance status, education level and marital status, we also investigated 15 community (census tract) SDOH using the American Community Survey. Because of the high correlation in the community SDOH, we developed the community-level indices representing different domains. Using Bayesian multilevel spatial models that account for the geographic dependency, we were able to simultaneously investigate the individual- and community-level SDOH that may impact HbA1C reduction.

**Results:** After accounting for the diabetes self-management education classes taken by the participants and their length of stay in the program, we found that older age at baseline, being married (compared to being widowed or divorced) and English speaking (compared to Spanish) were significantly associated with greater HbA1C reduction. Moreover, we found that the community level SDOH were also highly associated with HbA1C reduction. With every percentile rank decrease in the socioeconomic advantage index, we estimated an additional 0.018% reduction in HbA1C [95% CI (−0.028, −0.007)]. Besides the socioeconomic advantage index, urban core opportunity and immigrant's cohesion and accessibility indices were also statistically associated with HbA1C reduction.

**Conclusion:** To our knowledge, our study is the first to utilize Bayesian multilevel spatial models and simultaneously investigate both individual- and community-level SDOH in the context of diabetes management. Our findings suggest that community SDOH play an important role in diabetes control and management, and the need to consider community and neighborhood context in future interventions programs to maximize their overall effectiveness.

## Introduction

Diabetes has been a major health burden for Hispanic population. Nationally, Hispanics are more likely to be diagnosed with diabetes (1), have complications associated with diabetes (2), and die from diabetes (3). In Lower Rio Valley Grande (LGVR) in south Texas, a region that is historically underserved and socioeconomically disadvantaged, the prevalence of diabetes among Mexican American adults is about 30.7%, compared to 12.2% nationwide (4, 5). It is also an extremely poor area that is highly populated with Mexican Americans. Interventions on diabetes management considering the social determinants of health (SDOH) are scarce. In response, a coalition of local clinics and community-based organizations implemented a chronic-care management program, *Salud y Vida* (SyV), to support local residents with uncontrolled diabetes beginning in 2013.

Growing evidence suggests that SDOH not only play important roles in disease risks and outcomes, but also in treatment effectiveness, patient adherence, and overall health outcomes (6, 7). For example, in an older non-Hispanic male black population, patients with higher level of self-efficacy, social support, and medication adherence were more likely to achieve better glycemic control (8). Another study based on a Swedish registry found that higher educational levels and being married were significantly associated with lower hemoglobin A1C (HbA1C) at the follow-up (9). However, most studies only examined the individual-level SDOH (10), and ignored the community or neighborhood SDOH that may also serve as the potential risk factors of the diabetes development and management (11, 12).

This study focuses on identifying multi-dimensional SDOH, both at the individual and community level, that contribute to the HbA1C reduction of the participants from the *Salud y Vida* (SyV) program. By developing a Bayesian multilevel regression model, we assessed a comprehensive list of SDOH factors, while accounting for the clustering of participants from the same community and the potential geographic dependency of the communities. Incorporating both individual and community SDOH in the analysis provides a deeper understanding of diabetes management, and may inform the refinement of culturally tailored, cost-effective programs for patients with diabetes. Findings from our study will also provide key information for prioritizing SDOH related to improved diabetes outcomes among underserved Mexican Americans.

## Materials and Methods

### Study Population

SyV is an ongoing community-based intervention program that offers services such as diabetes self-management education classes (DSME), led by a professional multidisciplinary team and tailored to meet participants' individual needs. Details of the program can be found in Reininger et al. (13). Most participants had uncontrolled diabetes (HbA1C over 9%) at the time of enrollment (i.e., baseline). In this analysis, we included 1,923 participants that were recruited from one clinic between October 2013 and September 2018. The clinic is a federally qualified health center and primarily serves low-income families. By excluding re-enrolled records (enrolled more than once), we included 1,848 individuals with complete data as described below in the present study. The data collected include basic demographic information (e.g., age, gender), and individual SDOH such as education level, employment status, marital status, housing (e.g., home ownership), insurance status, and access to transportation and social support. HbA1C was measured at the baseline and quarterly throughout the duration of the program.

**Figure 1:**
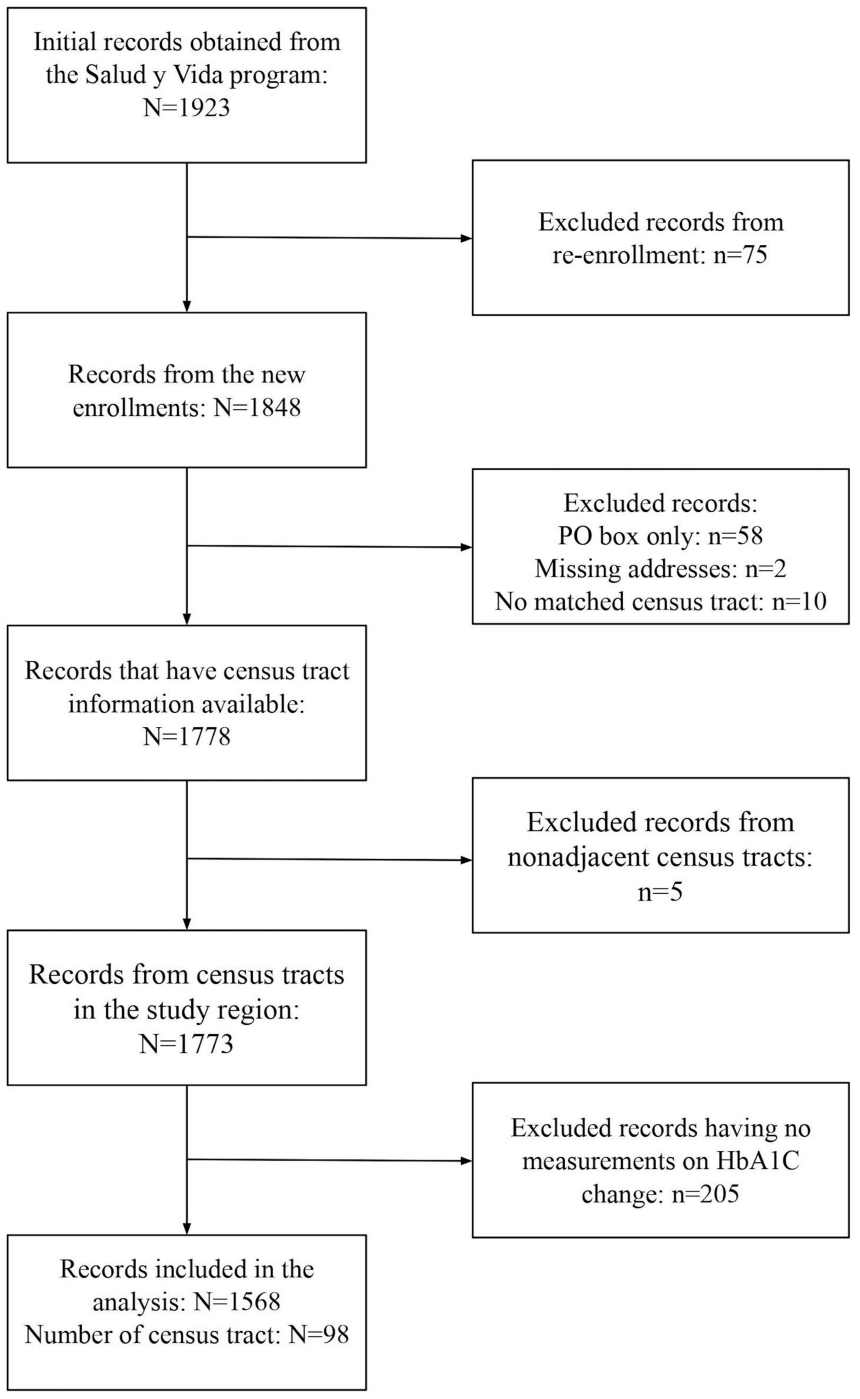
Data processing flow chart and sample size summaries for the Salud y Vida (SyV) data included in this study. Re-enrollment: enrolled more than once; new enrollment: enrolled once; PO box only: addresses with only PO box number; The addresses of participants were geocoded to obtain the census tract information, and only the records with census tract information from adjacent census tracts are included.

The community SDOH associated with each participant were measured at the census tract level. We first geocoded the participant's residential address and then obtained the corresponding census tract. We developed the geocoding algorithm using Google API (14) and Census API (15). For ambiguous or partial addresses, we manually checked Google map and made corrections. Participants with missing addresses, no matching census tract, or no measurement of HbA1C reduction were excluded from the analysis. The data processing procedure is presented in Figure 1. All geocoding and mapping was done in R (R Studio, Boston MA) (16).

The project was determined to be exempt by the University of Texas Health Science Center at Houston (UTHealth) Committee for the Protection of Human Subjects as study #HSC-SPH-20-0298.

### Census Tract Population and SDOH Data

Census tract population and SDOH data were obtained from the 2011 to 2015 American Community Survey (ACS) 5-Year Estimates (17). We selected 15 ACS variables roughly representing three SDOH domains: socioeconomic stability, demographic characteristics of disadvantaged groups, and housing and transportation. Socioeconomic stability included unemployment rate, poverty rate, per capita income, education attainment, and insurance status. Demographic characteristics included percentage of population aged under 18, population aged over 65, population with disability, limited English proficiency, racial minority, and single-parent households. Housing and transportation included percentage of renters, rent burden, crowded housing, and households with no vehicles. Per capita income was formatted as dollars and all other variables were formatted as proportions. Maps of the 15 ACS variables from 98 census tracts in the study region can be found in Supplementary eFigure 1.

### Constructing Tract-Level SDOH Indices

The tract-level SDOH variables are often highly correlated. In the 15 ACS variables we selected, some pairwise Pearson's correlation coefficients could be >0.3 (Supplementary eFigure 4). To address the potential multicollinearity problem in variable selection, we adopted the approach by Kolak et al. (18) where we used a principal component analysis (PCA) to create the multidimensional SDOH indices (see Supplementary Material eMethods for details), which represented four domains of community SDOH: socioeconomic advantage, mobility, urban core opportunity, and mixed immigration cohesion and accessibility (MICA), together explained 71.25% of the variance in the observed HbA1C reduction. We performed the analyses using the individual ACS variables, the constructed SDOH indices or both in the Bayesian multilevel models, and compared the results.

### Bayesian Spatial Multilevel Model

The Bayesian multilevel model we developed included a two-level hierarchical nesting structure, representing SyV participants (*n* = 1,568) clustered within census tracts (*n* = 98) (Figure 1). The outcome variable was the reduction of HbA1C measured as the difference between the last follow-up and the baseline. We assumed that the HbA1C reduction of the *i*th participant living in the *j*th census tract, denoted as *Y*_*ij*_, can be modeled as

$$y_{ij}=\beta_{0}+\beta_{i}{x_{ij}}^{T}+\gamma_{j}{z_{j}}^{T}+\varphi_{i}+\varepsilon_{ij},$$

where covariate vector **x**_**ij**_ represented the individual demographics and SDOH, and **z**_**j**_ represented the community SDOH, with corresponding regression coefficient vectors **β**_**i**_ and **γ**_**j**_. Error term ε_*ij*_ measured the within-participant residual and followed a normal distribution with mean zero and variance $\sigma_{\varepsilon }^{2}.$ We included a tract-level random effect φ_*i*_ which followed a multivariate normal distribution. If the communities were considered independent after accounting for the covariates, φ_*i*_ would be independently and identically distributed. Otherwise, we could consider certain dependency in φ_*i*_. Here we focused on the geographical dependency and considered three existing Bayesian spatial models for the random effect φ_*i*_: Besag, York, and Molliè (BYM) model (19), Besag's proper spatial model (20), and Leroux model (21). The best performing model was selected using conditional predictive ordinate (CPO) (22), widely applicable Bayesian information criterion (WAIC) (23), and deviance information criterion (DIC) (24). Details of the spatial model structure, priors used in the analysis, and the variable selection procedure are included in Supplementary—eMethods. The model with the lowest values of all three criteria was selected as our final model for inference. All analyses were conducted using R and R package INLA (25).

## Results

### Study Population Characteristics

The characteristics of the final 1,568 adults included in this analysis were summarized in Table 1. Most participants were female (65.7%), married (60.8%), speaking Spanish (66.2%), with an education level of 8th grade or below (40.7%) and without health insurance (78.3%). The average HbA1C (%) was 10.2 at the baseline and 8.7 at the last follow-up, with the average reduction of HbA1C (%) of 1.46. The average number of DSME classes the participants took were 5.4. At the census tract level, the number of participants ranged from 1 to 86 with an average of 16. The small sample size in some census tracts posed challenges in providing reliable estimates, which motivated us to adopt the Bayesian multilevel approach (26, 27). At census tract level, we observed substantial spatial variation in HbA1C reduction (Supplementary eFigures 2, 3), which further supported the use of multilevel modeling with spatial random effects.

**Table 1:** Characteristics of the SyV participants included in the analysis.

**Variable**	**Overall (*n* = 1,568)**
**HbA1C reduction**	
Mean (CV%)	1.46 (143.5%)
Median [Q1, Q3]	1.30 [0.100, 2.60]
**Age category**	
60 or greater	302 (19.3%)
Between 18 and 39	241 (15.4%)
Between 40 and 49	448 (28.6%)
Between 50 and 59	577 (36.8%)
**Gender**	
Male	538 (34.3%)
Female	1,030 (65.7%)
**Marital Status**	
Married	953 (60.8%)
Single	311 (19.8%)
Divorced	102 (6.5%)
Separated/widowed	175 (11.2%)
**Language**	
English	530 (33.8%)
Spanish	1,038 (66.2%)
**Education**	
8th grade or less	638 (40.7%)
Some high school	278 (17.7%)
High school graduate/GED	261 (16.6%)
Some college/college degree/graduate degree	241 (15.4%)
**Employment**	
Employed	415 (26.5%)
Disabled/retired	129 (8.2%)
Unemployed	675 (43.0%)
Other	220 (14.0%)
**Housing**	
Rent	475 (30.3%)
Own	714 (45.5%)
Other	146 (9.3%)
**Insurance Status**	
Yes	273 (17.4%)
No	1,228 (78.3%)
**DSME Class**	
Mean (CV%)	5.41 (79.9%)
Median [Q1, Q3]	6.00 [2.00, 7.00]
**Stay Days**	
Mean (CV%)	406 (33.0%)
Median [Q1, Q3]	387 [357, 461]
**Lack of Transportation**	
Yes	128 (8.2%)
No	1,406 (89.7%)
**Lack of Social Support**	
Yes	243 (15.5%)
No	1291 (82.3%)
**Coronary Heart Disease**	
Yes	93 (5.9%)
No	1428 (91.1%)
**High Blood Pressure**	
Yes	817 (52.1%)
No	701 (44.7%)

*All participants included in this study had uncontrolled diabetes (HbA1C over 9%) at baseline. HbA1C reduction is the difference of HbA1C measurement at last follow-up and the baseline*.

*CV, coefficient of variation; Q1, first quantile; Q3, third quantile; GED, certificate of high school equivalency; DSME, diabetes self-management education class*.

### Selecting Individual- and Community-Level SDOH

**Figure 2:**
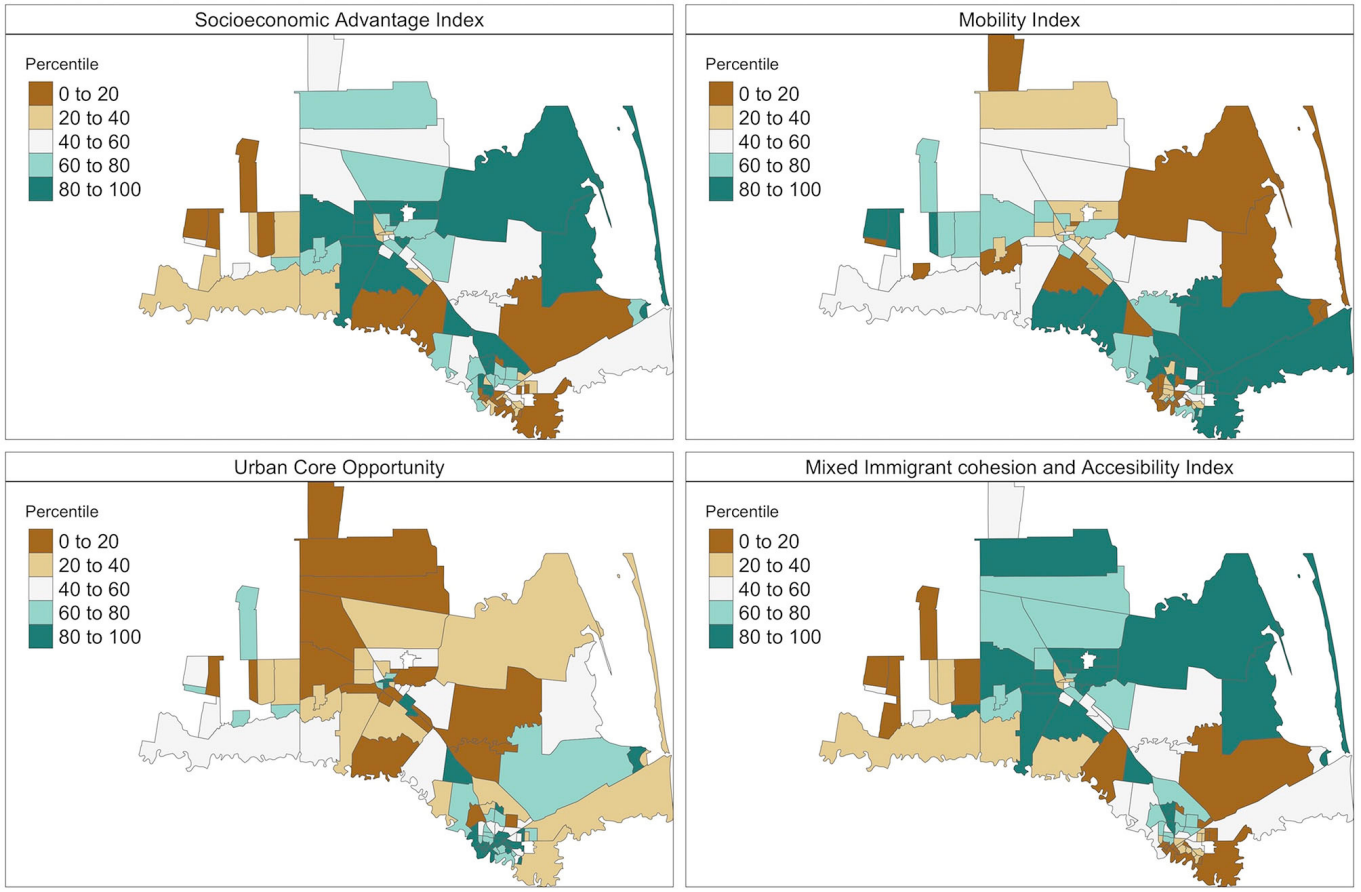
Maps of the social determinants of health (SDOH) indices. The four indices were created by applying principal component analysis on the 15 ACS variables. The presented indices are the ranked percentiles of the standardized SDOH indices within the study region, with higher percentiles (green shades) indicating advantage and lower percentiles (brown shades) indicating disadvantage.

To select the individual SDOH for the Bayesian multilevel models, we first fit a multivariable linear regression model that included all individual-level SDOH presented in Table 1, and then used an elastic net model to identify those with relatively high importance. The 15 tract-level ACS variables are summarized in Table 2. Compared with the US general population, the population in our study region had higher percentage of living in poverty, uninsured, with crowded housing, limited English proficiency, and lower education. The summary statistics of the four SDOH indices we constructed were also presented in Table 2. Instead of using the index values, we used the percentile rank of the index in multilevel analysis, as the latter had better interpretation. Maps of the tract SDOH indices presented in Figure 2 showed substantial spatial heterogeneity within the study region.

**Table 2:** Summary statistics for the 15 ACS variables in US and within study sample.

**Variable**	**US (*n* = 73,483)**	**In SyV (*n* = 98)**
**Disability**^**a**^**, %**		
Mean (CV%)	13.1 (44.8%)	14.2 (24.8%)
Median [Q1, Q3]	12.3 [8.90, 16.4]	14.4 [11.8, 16.6]
**No high school diploma**^**b**^**, %**		
Mean (CV%)	14.1 (79.6%)	38.8 (37.8%)
Median [Q1, Q3]	11.0 [6.00, 19.1]	39.8 [27.2, 51.8]
**Limited English**^**c**^**, %**		
Mean (CV%)	8.07 (136.6%)	30.5 (36.7%)
Median [Q1, Q3]	3.50 [1.10, 10.2]	30.0 [21.6, 40.1]
**Unemployed**^**d**^**, %**		
Mean (CV%)	5.45 (60.5%)	5.64 (39.8%)
Median [Q1, Q3]	4.80 [3.20, 6.90]	5.70 [4.05, 7.28]
**Uninsured**^**e**^**, %**		
Mean (CV%)	13.0 (64.7%)	32.4 (24.8%)
Median [Q1, Q3]	11.3 [6.70, 17.4]	35.0 [25.4, 37.7]
**Per capita income, $**		
Mean (CV%)	28,500 (52.9%)	14,200 (41.3%)
Median [Q1, Q3]	25,300 [19,100, 33,900]	12,200 [9,650, 18,000]
**Living poverty, %**		
Mean (CV%)	17.0 (77.7%)	37.0 (32.2%)
Median [Q1, Q3]	13.4 [7.20, 23.2]	37.7 [29.4, 43.3]
**Crowded housing**^**f**^**, %**		
Mean (CV%)	3.56 (149.5%)	13.3 (54.7%)
Median [Q1, Q3]	1.80 [0.600, 4.20]	12.3 [7.53, 17.9]
**Renters, %**		
Mean (CV%)	36.7 (61.9%)	34.4 (47.4%)
Median [Q1, Q3]	31.6 [18.8, 50.9]	31.3 [20.5, 44.0]
**No vehicle, %**		
Mean (CV%)	9.90 (126.7%)	9.10 (75.6%)
Median [Q1, Q3]	5.60 [2.70, 11.7]	7.70 [4.63, 12.0]
**Rent burden**^**g**^**, %**		
Mean (CV%)	50.1 (32.5%)	55.8 (25.2%)
Median [Q1, Q3]	50.5 [39.8, 60.9]	55.4 [46.2, 66.7]
**Aged under 18, %**		
Mean (CV%)	22.6 (29.7%)	31.6 (17.7%)
Median [Q1, Q3]	22.7 [19.0, 26.5]	31.5 [27.9, 35.2]
**Aged over 65, %**		
Mean (CV%)	14.8 (52.6%)	12.7 (39.3%)
Median [Q1, Q3]	14.0 [10.0, 18.2]	12.2 [9.43, 15.1]
**Racial minority**^**h**^**, %**		
Mean (CV%)	37.8 (81.1%)	90.5 (12.7%)
Median [Q1, Q3]	28.4 [11.6, 60.2]	94.4 [88.7, 97.4]
**Single parent**^**i**^**, %**		
Mean (CV%)	9.77 (67.9%)	15.0 (37.0%)
Median [Q1, Q3]	8.30 [5.10, 12.9]	15.2 [10.6, 19.3]
**Advantage index**^**j**^		
Median [Q1, Q3]	0.257 [−0.552, 0.764]	−0.277 [−0.834, 0.799]
**Mobility index**		
Median [Q1, Q3]	0.081 [−0.529, 0.638]	0.070 [−0.684, 0.634]
**Opportunity index**^**k**^		
Median [Q1, Q3]	−0.237 [−0.637, 0.376]	−0.161 [−0.700, 0.574]
**MICA index**^**l**^		
Median [Q1, Q3]	0.289 [−0.303, 0.666]	−0.058 [−0.826, 0.891]

*CV, coefficient of variation; Q1, first quantile; Q3, third quantile*.

*Data are estimates from the 2011 to 2015 American Survey 5-year estimates*.

a *Persons in the civilian non-institutionalized population*.

b *Persons aged 25 years or older*.

c *Persons aged 5 years and older*.

d *Civilians aged 16 years and older*.

e *Persons in the total civilian non-institutionalized population*.

f *Defined as occupied housing units consisting of more people than rooms*.

g *Renter paying more than 30% of their household income for rent*.

h *Defined as persons with the exception of white, non-Hispanic ancestry*.

i *Households with children <18 year*.

j *Socioeconomic advantage index*.

k *Urban core opportunity index*.

l *Mixed immigrant cohesion and accessibility index*.

### Bayesian Multilevel Spatial Models

The results of Bayesian multilevel spatial models were presented in Table 3, along with the results using the models considering individual SDOH only and community SDOH only, for comparison. The individual SDOH were selected after the elastic net model, and included baseline HbA1C, age, education level, marital status, length-of-stay in the program and the number of DSME classes attended. The community SDOH included the constructed four indices in rank percentiles. We investigated multilevel models assuming different spatial structures and selected the BYM model as the final model for inference as it outperformed the other models. Details of the analyses and comparison of different spatial models can be found in Supplementary Material eMethods.

**Table 3:** Results of Individual-level model, community-level model and Bayesian spatial multilevel model.

	Individual	Community	Multilevel BYM
	**Estimates (95% CI)**	**Estimates (95% CI)**	**Estimates (95% CI)**
**Baseline HbA1C**	0.70 (0.65, 0.76)	0.65 (0.60, 0.71)	0.70 (0.65, 0.75)
**Duration (<439 days)**	0.004 (0.003, 0.005)	–	0.004 (0.003, 0.005)
**Duration (≥439 days)**	0.001 (0, 0.001)	–	0.001 (0, 0.001)
**Language**			
English		Reference	
Spanish	−0.16 (−0.38,0.05)	–	−0.17 (−0.39, 0.05)
**Age**			
60 and greater		Reference	
Between 18 and 39	−0.57 (−0.87, −0.27)	–	−0.59 (−0.89, −0.29)
Between 40 and 49	−0.45 (−0.71, −0.20)	–	−0.44 (−0.70, −0.19)
Between 50 and 59	−0.33 (−0.57, −0.09)	–	−0.33 (−0.57, −0.08)
**Marital status**			
Married		Reference	
Divorced	−0.26 (−0.61, 0.09)	–	−0.29 (−0.64, 0.06)
Separated/widowed	−0.40 (−0.68, −0.12)	–	−0.44 (−0.72, −0.16)
Single	−0.16 (−0.39, 0.06)	–	−0.19 (−0.41, 0.04)
**Education**			
8th grade or less		Reference	
High school graduate	0.13 (−0.14, 0.40)	–	0.13 (−0.14, 0.39)
College/graduate degree	0.14 (−0.14, 0.42)	–	0.13 (−0.15, 0.41)
Some high school	0.20 (−0.04, 0.45)	–	0.20 (−0.05, 0.44)
**DSME class**	0.03 (0.01, 0.05)	–	0.03 (0.01, 0.05)
**Advantage rank**	–	−0.021 (−0.032, −0.01)	−0.018 (−0.028, −0.007)
**MICA rank**	–	0.023 (0.011, 0.035)	0.019 (0.007, 0.03)
**Mobility rank**	–	0.004 (0, 0.008)	0.004 (0, 0.007)
**Opportunity rank**	–	0.006 (0.001, 0.01)	0.005 (0.001, 0.009)
**CPO**	3,025.91	3,119.03	3,022.26
**WAIC**	6,051.81	6,238.06	6,044.52
**DIC**	6,050.73	6,237.65	6,043.66

*Individual-level model includes the individual variables selected after elastic net model. Community-level model includes the four SDOH indices as ranked percentiles. The multilevel BYM model includes both the individual and community level SDOH. Posterior mean is used for point estimate and posterior 95% credible interval is used as 95% CI. The model fit of the three models are included as CPO, WAIC, and DIC*.

The HbA1C reduction was statistically significantly associated with baseline HbA1C (0.699, 95% CI [0.647, 0.752]); participants with higher baseline HbA1C were likely to have greater HbA1C reduction. Age at baseline was also highly associated with HbA1C reduction, and the younger age groups tended to have smaller HbA1C reduction compared with the older age group (60 and above). Compared to participants who were married, participants who were separated or widowed had a significant smaller HbA1C reduction (−0.44, 95% CI [−0.72, −0.16]). Spanish-speaking participants had a lower HbA1C reduction compared to the English-speaking participants (−0.17, CI [−0.39, 0.052]). We observed no significant association between education level and the HbA1C reduction. Greater HbA1C reduction was associated with the number of DSME classes attended (0.03, 95% CI [0.01, 0.05]). We included a piecewise linear term to model the length of stay (in days), and the results showed that length of stay in the program was positively associated with the reduction of HbA1C (0.004, 95% CI [0.003, 0.005]), but only when the total length of stay was <439 days; after participants stayed longer than 439 days, there was no further reduction in HbA1C. Most of the 15 individual community-level SDOH were not significantly associated with HbA1C reduction, with the exception of crowded housing, which showed an inverse association with HbA1C reduction (−0.05, 95% CI [−0.08, −0.02], Supplementary eTable 3). Among the community SDOH indices, the advantage percentile rank was significantly associated with HbA1C reduction (−0.018, 95% CI [−0.028, −0.007]), suggesting that participants from higher SES tracts had worse improvement in diabetes control than lower SES tracts. MICA and urban core opportunity (opportunity) indices were also significantly associated with HbA1C reduction (0.005, 95% CI [0.001, 0.009] for MICA; 0.019, 95% CI [0.007, 0.03] for opportunity). No significant association was found with the mobility index percentile rank (0.004, 95% CI [0, 0.007]).

## Discussion

In this study, we identified the individual-level and community-level SDOH that were associated with HbA1C reduction in a diabetes management program, designed for a socioeconomically disadvantaged Mexican American population living on the US–Mexico border. Our analysis showed that the reduction in HbA1C between the baseline and the last follow-up significantly varied by numerous factors, at both individual and community levels. After accounting for program participation (i.e., time stayed in the program, and the number of DSME classes taken from the program) and baseline HbA1C level, individual factors included sociodemographic characteristics (i.e., baseline age, primary language speaking, marital status, education level) and community-level factors included socioeconomic advantage, urban core opportunity and immigration cohesion and accessibility.

We should note that our analysis is not trying to provide a comprehensive evaluation of the effectiveness of the SyV program, as it has been reported elsewhere (13). Rather, we aimed to investigate how different SDOH might contribute to the HbA1C reduction, *after* taking into account the participants' engagement in the program. We found that participants who attended more DSME classes had higher HbA1C reduction, which was consistent with the findings from Reininger et al. (13), where high engagement groups (≥2 visits to service provided by intervention program) showed greater HbA1C reduction over the study period, but low engagement groups (<2 visits) were not able to maintain the reduction in HbA1C. We also included the length of day as a way to account for engagement, and found that a longer stay in the program was also associated with a higher reduction in HbA1C; however, the association had a plateau effect and diminished after 439 days.

After considering individual's engagement in the program, we found that being married was associated with greater HbA1C reduction. This agreed with previous studies, as people who were married were more likely to have a higher level of diabetes-related lifestyle adaptation, less diabetes-related distress, and better quality of life (28). We also found that younger groups at baseline (<60 years) had a smaller HbA1C reduction compared with older age group (60 and above), also consistent with previous studies that individuals diagnosed with diabetes between 35 and 60 years old had worse glycemic control compared to those diagnosed at 65 years or older (29). The reasons why younger people exhibit poorer glycemic control could be manifold, as they may have a more severe form of the disease and/or a higher degree of insulin resistance (30), lower participation or program engagement (31), or face more competing work and family obligations that prevent lifestyle change (32, 33). Our findings suggest that the effectiveness of diabetes management varies across demographic groups. It is thus important to identify barriers that may prevent certain groups from fully benefiting from intervention programs like SyV.

At the community or neighborhood level, we had some unexpected findings that participants living in socioeconomically more advantaged census tracts exhibited smaller HbA1C reduction at the end of the program. This finding was opposite of previous studies showing that people living in neighborhoods with high socioeconomic status had better glycemic control (34). The inconsistency of the findings may be partly due to the lower prevalence of diabetes in the higher socioeconomic-advantage census tracts in the study sample, as we found a negative association between the baseline HbA1C and the socioeconomic advantage index. However, the inverse association between community socioeconomic advantage and HbA1C reduction persisted even after controlling for baseline HbA1C levels, suggesting that baseline severity of diabetes could not fully explain the observed results. Another possible interpretation might be that since the study populations are even more disadvantaged compared to the whole population in the study region, those disadvantaged individuals from wealthier census tracts may experience an increased level of depression and stress and in turn, had negative impacts on glycemic control. Finally, a previous study suggested people living in socioeconomically disadvantaged neighborhoods are more likely to use health care services than people from a more advantaged neighborhoods (35). Indeed, we saw that people from less advantaged communities on average participated in more classes (5.68 for first quartile of advantage index and 5.25 for fourth quartile of advantage index, Supplementary eTable 2) and stayed longer in the program (319 days for first quartile of advantage index and 295 days for fourth quartile of advantage index, Supplementary eTable 2).Therefore, a potentially higher engagement in the program among those living in more socioeconomically disadvantaged areas may have partially contributed greater HbA1C reduction in this group. Contrary to the inverse association observed for community socioeconomic advantage, we found that participants from census tracts with higher opportunity index rank or higher immigration cohesion and accessibility index rank had a greater HbA1C reduction. This was consistent with the previous study that social support and social cohesion had a significant positive influence on glycemic control (36). The mixed findings for different community indices suggested a complex and multidimensional impact of community context on diabetes control outcomes, and that future studies should investigate how multiple domains of community SDOH independently and collectively influence the effectiveness of health interventions.

To our knowledge, the study is the first to utilize Bayesian multilevel spatial models and simultaneously investigate both individual- and community-level SDOH in the context of diabetes management. Our analysis results suggested that both individual- and community-level SDOH were important factors with diabetes management and control. Solely relying on individual-level factors may overlook the neighborhood and environmental effects on individuals' lifestyles and decisions. Therefore, comprehensive approaches to diabetes control and management should not only target individual-level education and skill training but also include neighborhood context to improve the overall effectiveness. Intervention programs targeting behavior change at the community level could potentially improve diabetes-related health outcomes, and hence reduce health disparities in this disadvantaged Mexican American group. Our study has strength in several aspects. First, our study population is from a Mexican American population in an extremely poor area and with a high prevalence of diabetes and obesity, among which many have undiagnosed diabetes. This population is highly disadvantaged socioeconomically and understudied, and as such, our study provided the needed knowledge on SDOH and their impacts for this population on diabetes management. Second, though previous neighborhood SDOHs have been studied, they tend to focus only on one dimension (e.g., social economic status only), while our analysis included several different social dimensions, as well as including both individual and neighborhood level of SDOH together. Third, our Bayesian statistical model is a novel application in the context of SDOH and diabetes management. In addition, instead of only investigating the direction of the associations, we are *quantifying* the association of HbA1C reduction in relation to various SDOH domain. Such quantification could be very useful in the design of future diabetes management program and has high clinical relevance in personalized intervention. Our analysis also presented several limitations. First, though the data were collected longitudinally, we only investigated the difference in HbA1C reduction at the last follow up and the baseline, and therefore ignored the longitudinal HbA1C trajectory. This is indeed within our future plan to expand the Bayesian multilevel model presented here to model longitudinal diabetes control trajectories, as well as to investigate the potential difference of individual trajectory by neighborhood SDOH. As the number of individual and neighborhood SDOH increases, one may need to consider the issue of variable selection. In our analysis, we performed a priori process of variables selection using elastic net regression, but other approaches are available (37). Second, since we didn't have information on how long the participants have had diabetes, we were not able to assess the impact of duration of the disease on the effectives of the program. Third, we were not able to investigate the comorbidity of diabetes when assessing the effectiveness of diabetes management. Diabetes comorbidities, including depression, have been shown to lead to higher rates of complications in diabetes and disability, and may impact the diabetes control (38). Finally, measurements on lifestyle modification of the participants during the program, such as nutrition and physical activity, could be important factors affecting diabetes outcomes but were not included in our analysis.

To our knowledge, our study was the first to utilize Bayesian multilevel modeling with both individual- and community-level SDOH for diabetes management. The findings provide some explanation on the variation we see in HbA1C reduction from the participants, and shed some light on how to better design and implement future diabetes control and management programs. Multilevel intervention programs that are neighborhood-based and focused on SDOH are potentially effective in reducing uncontrolled diabetes among Mexican American populations.

## Data Availability Statement

The data that support the findings of this study are available from Salud y Vida program but restrictions apply to the availability of these data, which were used under license for the current study, and so are not publicly available. Data are, however, available from the authors upon reasonable request. Requests to access these datasets should be directed to BR, Belinda.M.Reininger@uth.tmc.edu.

## Ethics Statement

Written informed consent was obtained from the individual(s) for the publication of any potentially identifiable images or data included in this article.

## Author Contributions

CB and KZ analyzed the data, wrote the manuscript draft, and took the overall responsibility for the paper. BR, ML, and QX contributed significantly to the editing of the manuscripts. All authors contributed to the critical review of the paper and analytical results.

## Conflict of Interest

The authors declare that the research was conducted in the absence of any commercial or financial relationships that could be construed as a potential conflict of interest.
