# Supplementary material for: Individual and Community Social Determinants of Health Associated With Diabetes Management in a Mexican American Population
Source: Front Public Health. 2021 Feb 3;8:633340. doi: 10.3389/fpubh.2020.633340 (PMC7888279; doi:10.3389/fpubh.2020.633340)
Supplement: Supplementary file 1 [file Data_Sheet_1.docx]

**Supplementary materials for SDOH in the effectiveness of Salud y Vida** **program**

**eMethods.** Study population and community-level SDOH indices

**eFigure 1.** Map of the 15 selected ACS variables at census tract level

**eFigure 2.** Sample size of the Salud y Vida data at census tract level

**eFigure 3.** Average reduction in hemoglobin A1C (HbA1C) within study sample at census tract level

**eResults.** Principle component analysis, variable selection and Bayesian multilevel spatial modeling

**eFigure 4**. Correlation matrix of 15 ACS census-tract variables in 2015

**eFigure 5.** Results from PCA: scree plot and cumulative variance plot

**eFigure 6.** Variable importance measure plot

**eFigure 7.** Census-tracts included in this analysis and tracts spatial connectivity.

**eTable 1.** Variable loadings for the first 4 components

**eTable 2.** Summary statistics of the individual SDOH by socioeconomic advantage index quartiles

**eTable 3**. Results of linear regression model and Elastic net with individual level variables

**eTable 4.** Results of linear regression and Elastic net model with community-level variables

**eTable 5.** Results of linear regression model with three types of SDOH indices

**eTable 6.** Model fit comparison for different Bayesian multilevel models

**eTable 7**. Results of three Bayesian spatial multilevel models

**eReference.**

**eMethods.** Study population and community-level SDOH indices

Study population

Salud y Vida program was an intervention program designed to improve the health of the Hispanic population living in the Lower Rio Grande Valley with uncontrolled diabetes, through health education and social support. The program offered clinical services such as medication therapy management program and community service such as Diabetes Self-management Education classes (DSME), led by a professional multidiscipline team and tailored to meet participants’ individual needs. The DSME course offered in clinic or community consists of six classes on topics like nutrition, the importance of physical activity and general diabetes management. Participants of the program were adults who reside within the Hidalgo and Cameron Counties and had uncontrolled diabetes (HbA1c > 9.0) at baseline. Data collected from the program included the enrollment general information (e.g., baseline demographics of the participants), community health workers review survey, diabetes knowledge assessment, diabetes self-management assessment, diabetes medication adherence evaluation, Patient Health Questionaire-9, health status, medical history, behavioral health, and vital signs and lab results. The measurements on point of care such as HbA1C values, blood pressure, weight, and height were obtained at the baseline and every quarterly visit throughout the duration of the program.

## *Community-level SDOH indices*

## Principle component analysis (PCA) was conducted on the ACS data to create the multidimensional SDOH indices. In our analysis, the ACS variables were highly correlated, where many had a correlation coefficient greater than 0.3 (see the correlation matrix of the 15 variables in Appendix Table 1). The PCA is an unsupervised, non-parametric statistical technique for dimension reduction with better interpretability while minimizing information loss. In PCA, new uncorrelated components are created based on the variables to preserve as much as variances as possible^1^. The principal components can be used to reduce the factors included in data analysis or to produce index scores. We included all populated census tracts for the continental U.S. (n=73483) to preserve neighboring relationships across tracts. A multidimensional SDOH data matrix was developed using the 15 selected variables. All the variables were standardized (to Z-scores) as per capita income was measured at a different scale. Before PCA, the missing values are imputed by the regularized iterative PCA algorithm using R package missMDA^2^. The PCA was implemented using the single-value decomposition, which looks at the covariance and correlation among samples. The single-value decomposition method is preferred for numerical accuracy. Kaiser criteria were applied to determine the number of components to include after PCA, and only components with eigenvalues greater than 1 were retained to create SDOH indices. The scree plot and cumulative variance plot were used to identify the number of components. For each identified principal component, variables with a relatively large absolute value of variable loadings were considered as dominant in that component. For index score creation and interpretation, only the variables with loadings greater than 0.3 were retained in each component. An index score was produced for each of the identified components using the retained variables after being standardized and weighted by their loading coefficients. A composite SDOH index was constructed by summing up the weighted principal component scores.

**eFigure 1.** Map of the 15 selected ACS variables at census tract level


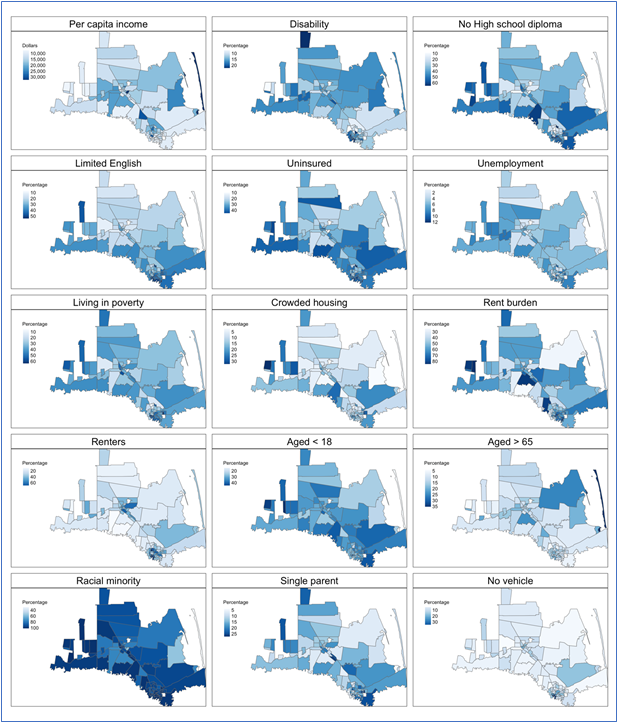


**eFigure 2.** Sample size of the Salud y vida data at census tract level


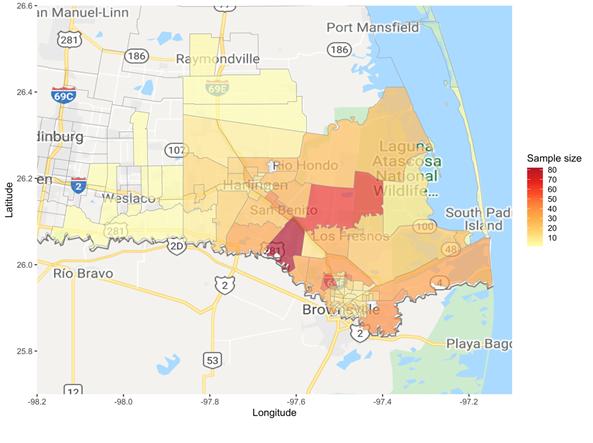


**eFigure 3.** Average reduction in hemoglobin A1C (HbA1C) within study sample at census tract level. with darker shaded areas indicating higher HbA1C reduction. There is substantial spatial variation observed across census tracts within the study area.


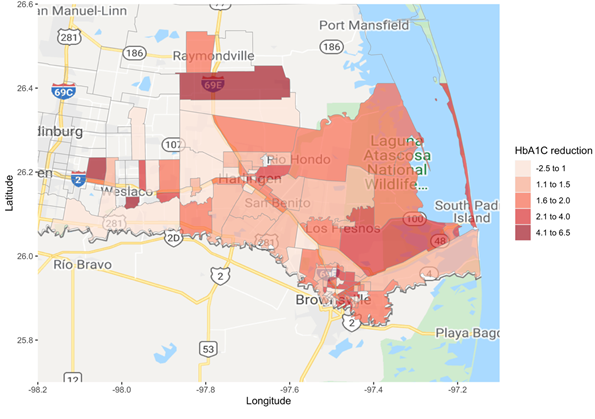


**eResults.** Principle component analysis, variable selection and Bayesian multilevel spatial modeling.

## *Principle component analysis*

The descriptive statistics of the 15 selected ACS variables are summarized in **Table 2** for all census tracts in the US and within the study sample. The median values of the census-tract ACS variables are generally higher within in the study region compared with those in the U.S., such as percentage of living in poverty, percentage of uninsured, percentage of crowded housing, percentage of racial minority, percentage of limited English proficiency and percentage of no high school diploma. The average percent of population living under poverty is 37.0% within-study sample, compared to 17.7% at the national level; the average percentage of people without high school diploma is 38.8% compared to 14.1% at the nation-level; the median of per capita income among SyV study sample is $14,200, much lower compared to $28,500 among the US. The maps of the 15 ACS variables are included in e**Figure 3**, in which substantial variations can be observed among the percentage of crowded housing, no high school diploma, unemployment, uninsured and limited English across all the census tracts within-study sample.

The first four PCA components had eigenvalues greater than 1.0 and thus were chosen to produce the standardized index scores (shown **eFigure 4a**). The amount of explained variance of the first four components is 71.25% in the 15 SDOH variables across all the U.S. census tracts (shown in **eFigure 4b**). The variables loadings of the four principal components are presented in e**Table 1**. Variables with relatively larger loadings are highlighted in bold for each component. The first principal component explained 40.54% of the total variance and was dominated by socioeconomic factors. The cardinality was adjusted so that advantaged areas get positive scores. The dominant variables include the proportion of no high school diploma (-0.34), uninsured (-0.29), below poverty (-0.32), single-parent households (-0.30), and per capita income (0.27). Therefore, we followed Kolak's paper and used the socioeconomic advantage index (SES index) for this component. The second principal component explained 13.2% of the total variance and was dominated by a higher proportion of older adults (aged 65 or older) and a higher proportion of people with disabilities. The proportions of older adults (-0.40) and people with disabilities (-0.59) were negatively related to the proportion of children (0.34). Therefore, we used the mobility index for this component following Kolak's et al (2020), with higher values suggesting better mobility. The third principal component explained 9.34% of the total variance and was dominated by per capita income (0.31), the proportion of renters (0.37), no vehicles (0.39), and children (-0.53). The areas included highly urbanized populations, with more opportunity and higher living costs and also with fewer children. Therefore, we used the urban core opportunity index following Kolak's et al (2020). The fourth principle component explained 8.17% of the total variance and was dominated by proportions of no high school diploma (-0.34), limited English proficiency (-0.32), crowded housing (-0.29), uninsured (-0.33) and older adults (-0.40). This index reflected several aspects of disadvantage, with higher proportions of immigrants and people who lacked access to social support. Therefore, mixed immigrant cohesion and accessibility index (MICA index) was used for this component following Kolak's et al (2020).

**eFigure 4**. Correlation matrix of 15 ACS census-tract variables in 2015

**eFigure 5.** Results from PCA: scree plot and cumulative variance plot


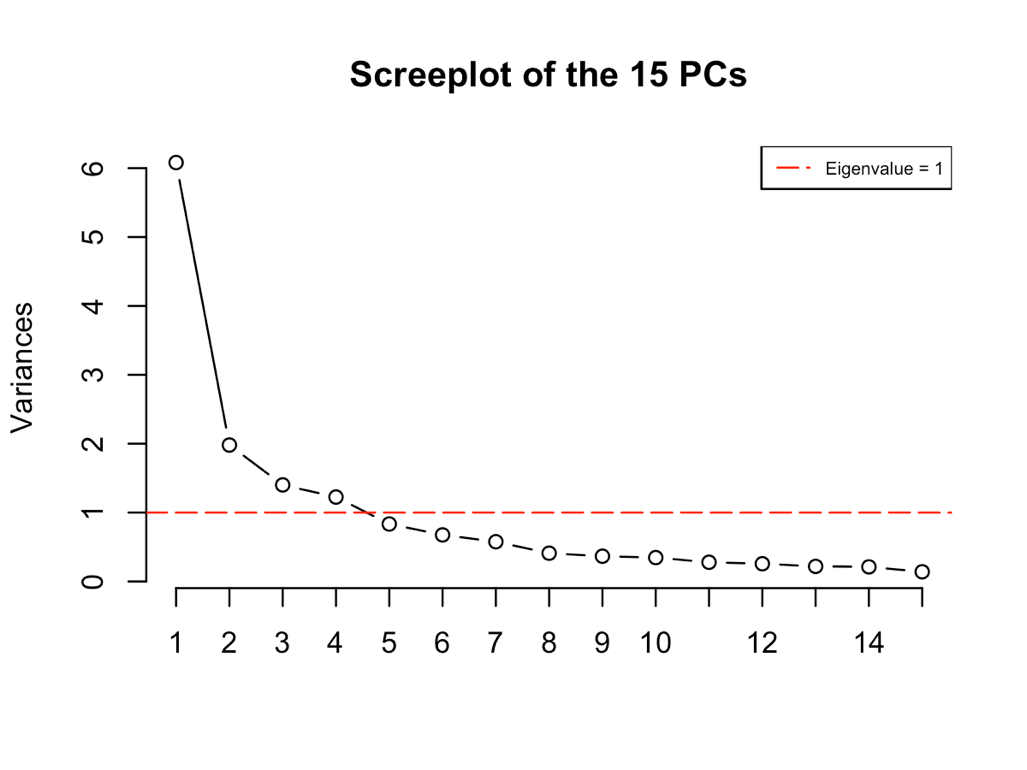


1. **Scree plot of the 15 principal components**


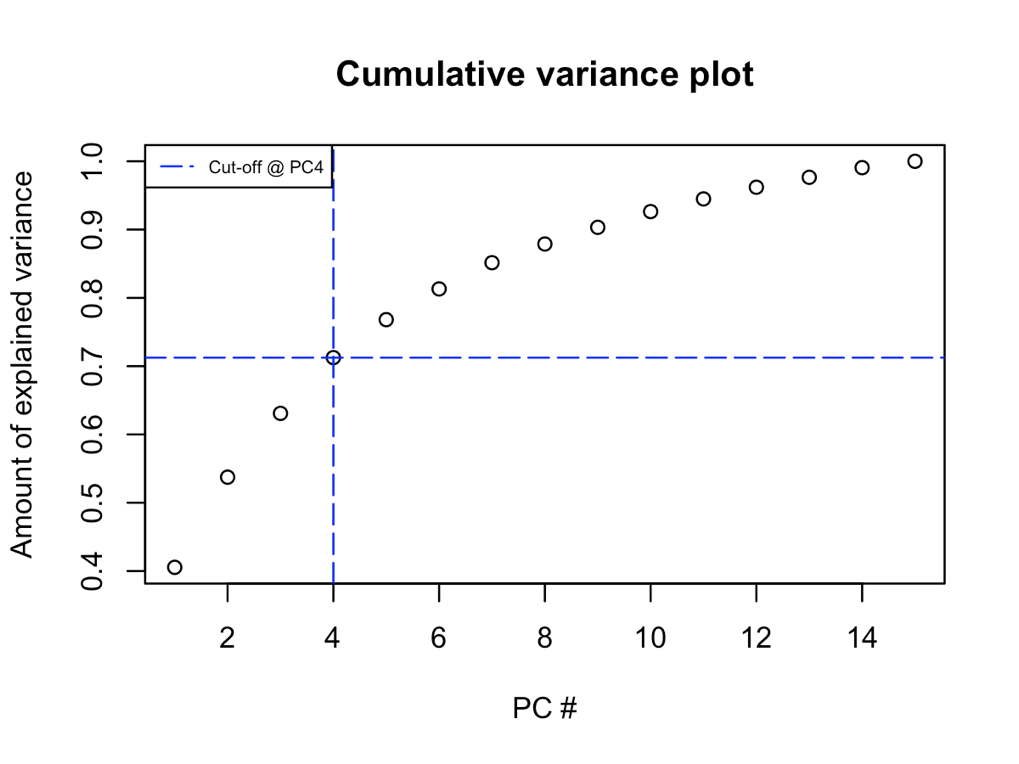


1. **Cumulative variance plot**

**eTable 1.** Variable loadings for the first 4 components

|  | PC1 | PC2 | PC3 | PC4 |
| --- | --- | --- | --- | --- |
| Disability | 0.0796 | **-0.5910** | 0.1679 | -0.2269 |
| No high school diploma | **0.3376** | -0.0204 | 0.0314 | **-0.3449** |
| Limited English Proficiency | 0.2595 | 0.2982 | **-0.3273** | **-0.3228** |
| Unemployed | 0.2537 | -0.1477 | 0.1766 | 0.2598 |
| Uninsured | **0.2938** | 0.0458 | 0.0103 | **-0.3257** |
| Per capita income | **-0.2695** | 0.1971 | **-0.3132** | 0.1540 |
| Living in poverty | **0.3189** | -0.2627 | 0.0370 | 0.1354 |
| Crowded housing | **0.2817** | 0.2587 | -0.1740 | **-0.2877** |
| Renters | **0.2714** | -0.0618 | **-0.3719** | **0.3258** |
| No vehicle | 0.2239 | -0.2116 | **-0.3876** | **0.3294** |
| Rent burden | 0.1847 | -0.1638 | -0.0530 | 0.0217 |
| Age under 18 | 0.1546 | **0.3387** | **0.5333** | 0.0477 |
| Age over 65 | -0.1763 | **-0.4007** | -0.1422 | **-0.3970** |
| Racial minority | **0.3236** | 0.1093 | -0.1123 | 0.0532 |
| Single parent | **0.3007** | 0.0019 | **0.3068** | 0.2217 |

**eTable2. Summary statistics of the individual SDOH by socioeconomic advantage index quartiles**

| Variable | First quartile (n=398) | Second quartile (n=375) | | Third quartile (n=402) | Fourth quartile (n=378) |
| --- | --- | --- | --- | --- | --- |
| HbA1C Reduction |  | |  |  |  |
| Mean (CV%) | 1.51 (141.3%) | | 1.55 (134.6%) | 1.39 (155.3%) | 1.40 (144.8%) |
| Median [Q1, Q3] | 1.25 [0.200, 2.60] | | 1.30 [0.200, 2.70] | 1.25 [0.00, 2.50] | 1.30 [0.200, 2.50] |
| Baseline HbA1C |  | |  |  |  |
| Mean (CV%) | 10.2 (16.0%) | | 10.2 (15.4%) | 10.2 (16.8%) | 10.2 (15.9%) |
| Median [Q1, Q3] | 9.90 [8.90, 11.2] | | 9.90 [9.00, 11.5] | 10.0 [8.80, 11.4] | 10.0 [8.80, 11.2] |
| Age Category |  | |  |  |  |
| 60 Or Greater | 71 (17.8%) | | 74 (19.7%) | 79 (19.7%) | 75 (19.8%) |
| Between 18 And 39 | 73 (18.3%) | | 54 (14.4%) | 54 (13.4%) | 59 (15.6%) |
| Between 40 And 49 | 113 (28.4%) | | 108 (28.8%) | 119 (29.6%) | 105 (27.8%) |
| Between 50 And 59 | 141 (35.4%) | | 139 (37.1%) | 150 (37.3%) | 139 (36.8%) |
| Gender |  | |  |  |  |
| Female | 272 (68.3%) | | 235 (62.7%) | 272 (67.7%) | 245 (64.8%) |
| Male | 126 (31.7%) | | 140 (37.3%) | 130 (32.3%) | 133 (35.2%) |
| Marital Status |  | |  |  |  |
| Divorced | 20 (5.0%) | | 26 (6.9%) | 26 (6.5%) | 29 (7.7%) |
| Married | 248 (62.3%) | | 221 (58.9%) | 242 (60.2%) | 230 (60.8%) |
| Separated/Widowed | 43 (10.8%) | | 46 (12.3%) | 47 (11.7%) | 38 (10.1%) |
| Single | 78 (19.6%) | | 76 (20.3%) | 78 (19.4%) | 78 (20.6%) |
| Language |  | |  |  |  |
| English | 80 (20.1%) | | 133 (35.5%) | 154 (38.3%) | 161 (42.6%) |
| Spanish | 318 (79.9%) | | 242 (64.5%) | 248 (61.7%) | 217 (57.4%) |
| Education |  | |  |  |  |
| 8th Grade or Less | 188 (47.2%) | | 148 (39.5%) | 160 (39.8%) | 134 (35.4%) |
| High School Graduate/Ged | 62 (15.6%) | | 65 (17.3%) | 66 (16.4%) | 67 (17.7%) |
| Some College/College Degree/Graduate Degree | 32 (8.0%) | | 63 (16.8%) | 73 (18.2%) | 71 (18.8%) |
| Some High School | 68 (17.1%) | | 72 (19.2%) | 67 (16.7%) | 70 (18.5%) |
| Employment |  | |  |  |  |
| Disabled/Retired | 26 (6.5%) | | 34 (9.1%) | 35 (8.7%) | 33 (8.7%) |
| Employed | 94 (23.6%) | | 102 (27.2%) | 106 (26.4%) | 106 (28.0%) |
| Other | 57 (14.3%) | | 55 (14.7%) | 55 (13.7%) | 52 (13.8%) |
| Unemployed | 181 (45.5%) | | 161 (42.9%) | 171 (42.5%) | 158 (41.8%) |
| Housing |  | |  |  |  |
| Other | 36 (9.0%) | | 41 (10.9%) | 38 (9.5%) | 30 (7.9%) |
| Own | 176 (44.2%) | | 177 (47.2%) | 177 (44.0%) | 179 (47.4%) |
| Rent | 110 (27.6%) | | 116 (30.9%) | 130 (32.3%) | 114 (30.2%) |
| Insurance Status |  | |  |  |  |
| No | 319 (80.2%) | | 287 (76.5%) | 317 (78.9%) | 295 (78.0%) |
| Yes | 58 (14.6%) | | 75 (20.0%) | 70 (17.4%) | 66 (17.5%) |
| DSME Class |  | |  |  |  |
| Mean (CV%) | 5.68 (78.0%) | | 5.48 (73.1%) | 5.20 (84.0%) | 5.25 (86.0%) |
| Median [Q1, Q3] | 6.00 [3.00, 7.00] | | 6.00 [2.50, 7.00] | 6.00 [2.00, 7.00] | 6.00 [2.00, 7.00] |
| Stay Days |  | |  |  |  |
| Mean (CV%) | 409 (34.1%) | | 406 (32.5%) | 411 (32.4%) | 397 (33.1%) |
| Median [Q1, Q3] | 392 [358, 458] | | 384 [357, 462] | 397 [357, 465] | 384 [357, 462] |
| Lack of Transportation |  | |  |  |  |
| No | 349 (87.7%) | | 338 (90.1%) | 362 (90.0%) | 344 (91.0%) |
| Yes | 39 (9.8%) | | 28 (7.5%) | 32 (8.0%) | 27 (7.1%) |
| Lack of Social Support |  | |  |  |  |
| No | 313 (78.6%) | | 306 (81.6%) | 342 (85.1%) | 318 (84.1%) |
| Yes | 75 (18.8%) | | 60 (16.0%) | 52 (12.9%) | 53 (14.0%) |
| Coronary Heart Disease |  | |  |  |  |
| No | 369 (92.7%) | | 335 (89.3%) | 364 (90.5%) | 346 (91.5%) |
| Yes | 16 (4.0%) | | 32 (8.5%) | 23 (5.7%) | 21 (5.6%) |
| High Blood Pressure |  | |  |  |  |
| No | 195 (49.0%) | | 157 (41.9%) | 166 (41.3%) | 178 (47.1%) |
| Yes | 190 (47.7%) | | 207 (55.2%) | 222 (55.2%) | 189 (50.0%) |

*Variable selection*

*Individual-level regression model*:

The individual-level regression model only included the individual-level SDOH. Linear regression was performed including all the selected characteristics with HbA1C reduction as an outcome variable. The elastic net model was applied to select statistically significant variables. Elastic net is ideal when the variables form groups that contain strongly correlated independent variables. These individual-level covariates included baseline HbA1C, age (18 to 39, 40 to 49, 50 to 59, or 60 or greater), gender (female or male), education level (8th grade or less, some high school, high school graduate, some college or college degree or graduate degree), employment status (employed, unemployed, disabled or retired, other), marital status (married, divorced, separated or widowed, single), housing (own or rent), insurance status (yes or no), diabetes-management education (number of classes taken), access to transportation and social support (yes or no), history of high blood pressure and coronary heart disease (yes or no).

*Community-level regression model*:

At the community level, the regression model was performed with the census-tract covariates only and the HbA1C reduction as an outcome variable. We considered different approaches to model their effects. We first included 15 individual ACS variables and used an elastic net for variable selection. We then considered the SDOH indices as the covariates in the model and included three ways of indices for comparison: the raw index, standardized index, and the percentile ranked index within the study sample. We chose to include the percentile ranked SDOH indices in the multilevel modeling due to its easy interpretation.

**eFigure 6.** Variable importance measure plot for individual-level elastic net

*Bayesian multilevel spatial model*

The three spatial models are Besag, York^3^, and Molliè (BYM) model^4^, Besag’s proper spatial model, and Leroux model^5^. The BYM model assumes two latent random effects: an intrinsic autoregressive (ICAR) random effect $v_{j}$ and a Gaussian iid random effect $\mu_{j}$. The ICAR model incorporates the spatial dependency by constructing j by k adjacency matrix $w_{jk}$, where 1 was assigned if two areas share the same geographic boundary and 0 otherwise. The census tracts included in the analysis and the tracts spatial connectivity are shown in **eFigure 7**. BYM model assumes convolution prior for the random effects:

$$\varphi_{j}=\mu_{j}+v_{j}，$$

$$\mu_{j}\sim N\left( 0, \delta_{\mu}^{2} \right),$$

$$v_{j}|v_{\neq j} \sim N\left( \frac{1}{\sum_{k} w_{jk}}\sum_{k} w_{jk}v_{k}, \frac{\sigma_{v}^{2}}{\sum_{k} w_{jk}} \right).$$

The Besag's proper spatial model is a variation of the BYM model by constructing a non-singular precision matrix:

$$\varphi_{j}|\varphi_{\neq j} \sim N\left( \frac{\rho}{\sum_{k} w_{jk}}\sum_{k} w_{jk}\varphi_{k}, \frac{\sigma_{\varphi}^{2}}{\sum_{k} w_{jk}} \right).$$

Leroux is another variation of the BYM model and only requires a single set of random effects, where

$$\varphi_{j}|\varphi_{\neq j} \sim N\left. \left\{ \frac{\rho\sum_{k=1, k\neq j}^{n} w_{jk}\varphi_{k}}{\rho\sum_{j=1, j\neq i}^{n} w_{ij}+1-\rho} \right., \frac{\sigma_{\varphi}^{2}}{\rho\sum_{k=1,k\neq j}^{n} w_{jk}+1-\rho} \right\}.$$

The parameter $\rho$ quantified the degree of spatial correlation between the spatial random effects$\varphi_{j}$, with $\rho=0$ corresponding to independence, and $\rho=1$ representing strong spatial correlation throughout the region. Residual variation not explained by spatial correlation was captured by the variance parameter $\sigma_{\varphi}^{2}$. We fit all Bayesian models using R (R Studio, Boston MA) and R package INLA^6^. The precisions for the intercept, fixed effects, and random effects are assigned the default priors in R-INLA, where $\log\left( 1/\sigma^{2} \right)\sim log-gamma (1, 0.001)$.

**eFigure 7.** Census tracts included in this analysis and tracts spatial connectivity.


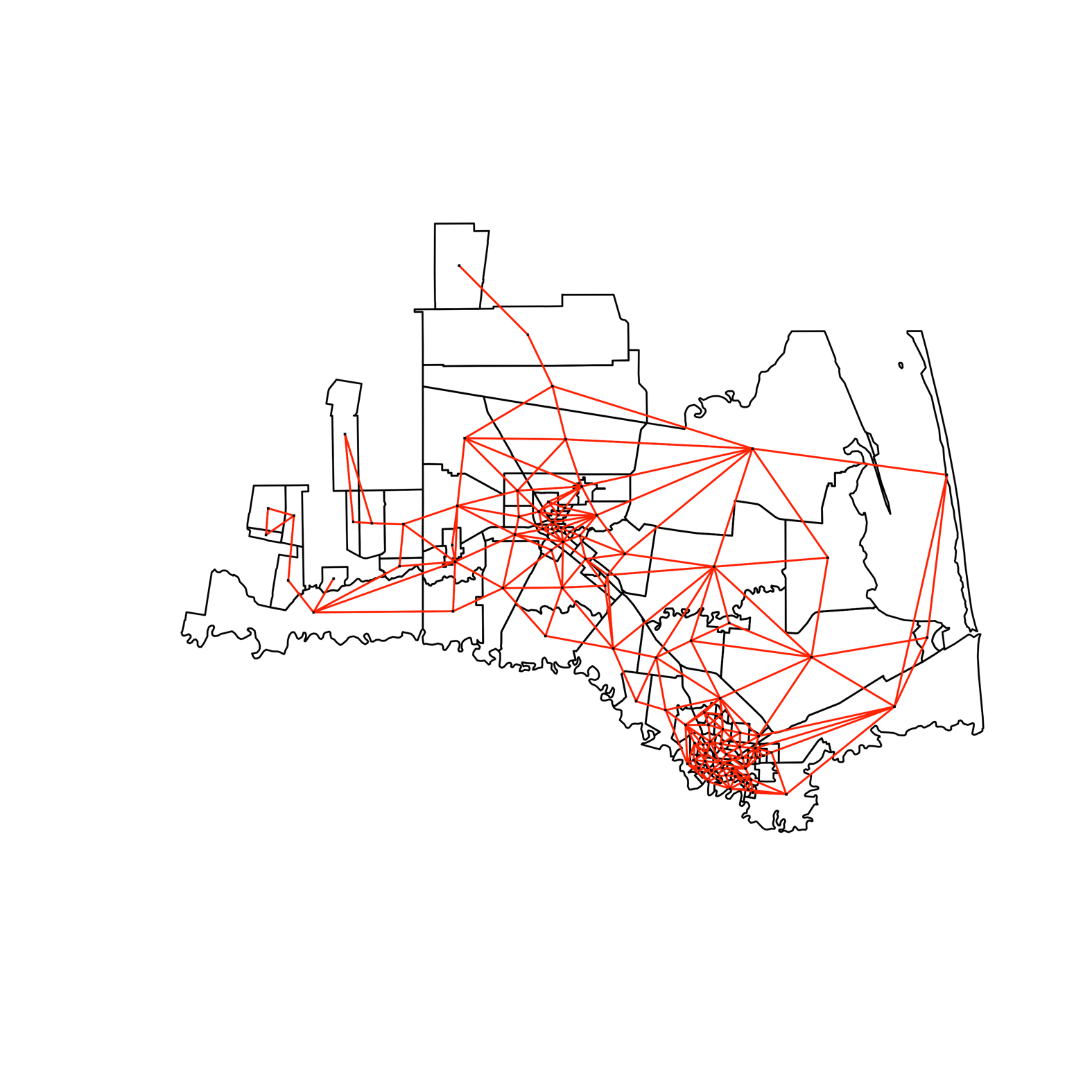


**eTable 3.** Results of linear regression model and Elastic net with individual level variables

|  | HbA1C Reduction | | |  | |
| --- | --- | --- | --- | --- | --- |
|  | **Linear Regression** | | | **Elastic Net** | |
| *Predictors* | *Estimates* | *CI* | *P value* | | *Estimates* |
| (Intercept) | -6.495 | (-7.385, -5.604) | **<0.001** | 1.562 | |
| Baseline HbA1C | 0.736 | (0.676, 0.795) | **<0.001** | 1.188 | |
| Duration (<439 days) | 0.004 | (0.002, 0.005) | **<0.001** | 0.541 | |
| Duration (>=439 days) | 0.000 | (-0.000, 0.001) | 0.389 | - | |
| DSME classes | 0.038 | (0.015, 0.060) | **0.001** | 0.163 | |
| Gender |  |  |  |  | |
| Female |  | Reference |  |  | |
| Male | 0.054 | (-0.162, 0.271) | 0.622 | 0.009 | |
| Age |  |  |  |  | |
| 60 and greater |  | Reference |  |  | |
| Between 18 and 39 | -0.746 | (-1.122, -0.370) | **<0.001** | -0.188 | |
| Between 40 and 49 | -0.629 | (-0.941, -0.318) | **<0.001** | -0.201 | |
| Between 50 and 59 | -0.476 | (-0.760, -0.191) | **0.001** | -0.150 | |
| Language |  |  |  |  | |
| English |  | Reference |  |  | |
| Spanish | -0.154 | (-0.408, 0.099) | 0.232 | -0.060 | |
| Education |  |  |  |  | |
| 8^th^ grade or less |  | Reference |  |  | |
| High school graduate/GED | 0.126 | (-0.184, 0.436) | 0.425 | 0.021 | |
| College /graduate degree | 0.050 | (-0.271, 0.371) | 0.760 | - | |
| Some high school | 0.225 | (-0.053, 0.503) | 0.113 | 0.059 | |
| Employment |  |  |  |  | |
| Employed |  | Reference |  |  | |
| Disabled/ Retired | 0.029 | (-0.378, 0.437) | 0.887 | - | |
| Other | 0.309 | (0.001, 0.617) | **0.049** | 0.073 | |
| Unemployed | 0.097 | (-0.141, 0.335) | 0.424 | 0.020 | |
| Marital Status |  |  |  |  | |
| Married |  |  |  |  | |
| Divorced | -0.272 | (-0.662, 0.119) | 0.173 | -0.039 | |
| Separated/widowed | -0.393 | (-0.717, -0.068) | **0.018** | -0.090 | |
| Single | -0.277 | (-0.541, -0.014) | **0.039** | -0.070 | |
| Lack of transportation |  |  |  |  | |
| No |  | Reference |  |  | |
| Yes | 0.153 | (-0.205, 0.512) | 0.401 | 0.024 | |
| Lack of social support |  |  |  |  | |
| No |  | Reference |  |  | |
| Yes | 0.116 | (-0.156, 0.388) | 0.404 | 0.025 | |
| Insurance |  |  |  |  | |
| Yes |  | Reference |  |  | |
| No | -0.082 | (-0.348, 0.185) | 0.547 | -0.012 | |
| Housing |  |  |  |  | |
| Own |  | Reference |  |  | |
| Other | 0.125 | (-0.217, 0.468) | 0.473 | 0.008 | |
| Rent | 0.201 | (-0.016, 0.418) | 0.069 | 0.056 | |
| Coronary heart disease |  |  |  |  | |
| No |  | Reference |  |  | |
| Yes | -0.292 | (-0.696, 0.111) | 0.156 | -0.046 | |
| High blood pressure |  |  |  |  | |
| No |  | Reference |  |  | |
| Yes | -0.043 | (-0.252, 0.166) | 0.688 | - | |
| Observations | 1188 | | | 1188 | |
| R^2^ / R^2^ adjusted | 0.390 / 0.377 | | |  | |
|  |  |  |  |  |  |

**eTable 4.** Results of linear regression and Elastic net model with contexture variables

|  | HbA1C Difference | | |  |
| --- | --- | --- | --- | --- |
|  | **Linear Regression** | | | **Elastic Net** |
| *Predictors* | *Estimates* | *CI* | *p* | *Estimates* |
| (Intercept) | -4.186 | (-7.954, -0.418) | **0.029** | 1.471 |
| Baseline HbA1C | 0.653 | (0.597, 0.708) | **<0.001** | 1.039 |
| Disability, % | -0.018 | (-0.060, 0.025) | 0.416 | -0.053 |
| No High School diploma, % | -0.010 | (-0.029, 0.008) | 0.273 | - |
| Limited English, % | 0.019 | (-0.003, 0.041) | 0.086 | 0.049 |
| Per capita income, $ | -0.005 | (-0.010, 0.000) | 0.074 | -0.110 |
| Uninsured, % | 0.004 | (-0.023, 0.032) | 0.766 | - |
| Unemployed, % | -0.029 | (-0.084, 0.026) | 0.300 | -0.026 |
| Living in poverty, % | 0.014 | (-0.007, 0.034) | 0.184 | 0.075 |
| Crowded housing, % | -0.049 | (-0.080, -0.019) | **0.001** | -0.179 |
| Rent burden, % | -0.002 | (-0.010, 0.006) | 0.591 | - |
| Renters, % | 0.004 | (-0.006, 0.014) | 0.456 | 0.044 |
| Aged under 18, % | 0.024 | (-0.020, 0.068) | 0.286 | - |
| Aged over 65, % | 0.016 | (-0.025, 0.058) | 0.439 | - |
| Racial minority, % | -0.012 | (-0.032, 0.007) | 0.218 | -0.028 |
| Single parent, % | 0.010 | (-0.015, 0.036) | 0.421 | 0.048 |
| No vehicle, % | -0.007 | (-0.031, 0.017) | 0.554 | - |
| Observations | 1551 | | | 1551 |
| R^2^ / R^2^ adjusted | 0.274 / 0.266 | | |  |

**eTable 5.** Results of linear regression model with three types of SDOH indices

|  | HbA1C Difference | | | |  | | |  |
| --- | --- | --- | --- | --- | --- | --- | --- | --- |
|  | **Scaled Index Percentile Rank** | | | | **Raw Index** | | |  |
| *Predictors* | *Estimates* | *P value* | *Estimates* | *P value* | | *Estimates* | *P value* |  |
| (Intercept) | -5.188 | **<0.001** | -5.747 | **<0.001** | | -5.462 | **<0.001** |  |
| Baseline HbA1C | 0.652 | **<0.001** | 0.651 | **<0.001** | | 0.652 | **<0.001** |  |
| SES Index | -0.595 | **<0.001** | -0.021 | **<0.001** | | -0.526 | **<0.001** |  |
| Mobility Index | 0.090 | 0.147 | 0.004 | 0.053 | | 0.125 | 0.147 |  |
| MICA Index | 0.610 | **0.001** | 0.023 | **<0.001** | | 0.460 | **0.001** |  |
| Opportunity Index | 0.138 | 0.049 | 0.006 | **0.008** | | 0.295 | **0.049** |  |
| Observations rank | 1551 1551 | | | | 1551 | | |  |
| R^2^ / R^2^ adjusted | 0.261 / 0.259 0.262 / 0.260 | | | | 0.261 / 0.259 | | |  |
|  |  |  |  |  |  |  |  |  |

**eTable 6.** Model fit for difference Bayesian multilevel models. A null model with no covariates and a random intercept with only individual-level covariates were also included for comparison. Compare to the null model with only a normal random effect for each census tract, the random intercept model reduced the DIC by 657.05 by incorporating the individual-level covariates. The multilevel IID model assuming no spatial independence further reduced the DIC by 6.66, indicating that incorporating community-level random effects did improve the model fit. However, based on the three model fit criteria, multilevel models incorporating spatial dependency (Multilevel BYM, Multilevel Besag proper, and Multilevel Leroux model) didn't further improve model fit compared to the IID model.

| Model | CPO | WAIC | DIC |
| --- | --- | --- | --- |
| Null model | 3354.13 | 6708.25 | 6707.77 |
| Random intercept model (Individual level only) | 3025.91 | 6051.8 | 6050.72 |
| Multilevel IID model | 3022.55 | 6045.09 | 6044.06 |
| Multilevel BYM | 3022.26 | 6044.52 | 6043.66 |
| Multilevel Besagproper | 3022.5 | 6044.99 | 6044.13 |
| Multilevel Leroux | 3022.51 | 6045.02 | 6044.18 |

**eTable 7**. Results of three Bayesian spatial multilevel models

|  |  | | HbA1C Difference | | |  |
| --- | --- | --- | --- | --- | --- | --- |
|  | **BYM** | **Besag Proper** | | **Leroux** | | |
| *Predictors* | *Estimates (CI)* | *Estimates (CI)* | | *Estimates (CI)* | | |
| (Intercept) | -6.833 (-7.688, -5.979) | -6.832 (-7.683, -5.981) | | -6.831 (-7.684, -5.98) | | |
| Baseline HbA1C | 0.699 (0.647, 0.752) | 0.699 (0.647, 0.751) | | 0.699 (0.647, 0.752) | | |
| Duration (<439 days) | 0.004 (0.003, 0.005) | 0.004 (0.003, 0.005) | | 0.004 (0.003, 0.005) | | |
| Duration (>=439 days) | 0.001 (0, 0.001) | 0.001 (0, 0.001) | | | 0.001 (0, 0.001) | |
| Language |  |  | | |  | |
| English |  | Reference | | |  | |
| Spanish | -0.166 (-0.385, 0.052) | -0.166 (-0.384, 0.052) | | | -0.166 (-0.384, 0.053) | |
| Age |  |  | | |  | |
| 60 and greater |  | Reference | | |  | |
| Between 18 and 39 | -0.588 (-0.891, -0.285) | -0.588 (-0.891, -0.286) | | | -0.588 (-0.892, -0.285) | |
| Between 40 and 49 | -0.443 (-0.701, -0.186) | -0.444 (-0.701, -0.187) | | | -0.444 (-0.701, -0.187) | |
| Between 50 and 59 | -0.325 (-0.566, -0.084) | -0.325 (-0.566, -0.084) | | | -0.325 (-0.566, -0.084) | |
| Marital Status |  |  | | |  | |
| Married |  | Reference | | |  | |
| Divorced | -0.287 (-0.636, 0.061) | -0.288 (-0.636, 0.06) | | | -0.288 (-0.636, 0.06) | |
| Separated/ Widowed | -0.437 (-0.717, -0.157) | -0.437 (-0.717, -0.157) | | | -0.437 (-0.717, -0.157) | |
| Single | -0.186 (-0.408, 0.035) | -0.186 (-0.407, 0.035) | | | -0.186 (-0.408, 0.036) | |
| Education |  |  | | |  | |
| 8^th^ grade or less |  | Reference | | |  | |
| High school graduate | 0.127 (-0.139, 0.392) | 0.127 (-0.138, 0.391) | | | 0.126 (-0.139, 0.392) | |
| College/ Graduate degree | 0.132 (-0.147, 0.412) | 0.132 (-0.147, 0.411) | | | 0.132 (-0.147, 0.412) | |
| Some high school | 0.197 (-0.048, 0.441) | 0.198 (-0.047, 0.442) | | | 0.198 (-0.047, 0.442) | |
| DSME class | 0.031 (0.011, 0.051) | 0.031 (0.011, 0.051) | | | 0.031 (0.011, 0.051) | |
| SES rank | -0.018 (-0.028, -0.007) | -0.018 (-0.028, -0.007) | | | -0.018 (-0.028, -0.007) | |
| MICA rank | 0.019 (0.007, 0.03) | 0.019 (0.007, 0.03) | | | 0.019 (0.007, 0.03) | |
| Mobility rank | 0.004 (0, 0.007) | 0.004 (0, 0.007) | | | 0.004 (0, 0.007) | |
| Opportunity rank | 0.005 (0.001, 0.009) | 0.005 (0.001, 0.009) | | | 0.005 (0.001, 0.009) | |
|  |  |  |  |  |  |  |

**eReference**

1. Jolliffe IT, Cadima J. Principal component analysis: a review and recent developments. *Philos Trans R Soc Math Phys Eng Sci*. 2016;374(2065):20150202. doi:10.1098/rsta.2015.0202

2. Josse J, Husson F. missMDA: A Package for Handling Missing Values in Multivariate Data Analysis. *J Stat Softw*. 2016;70(1):1-31. doi:10.18637/jss.v070.i01

3. Besag J, York J, Mollié A. Bayesian image restoration, with two applications in spatial statistics. *Ann Inst Stat Math*. 1991;43(1):1-20. doi:10.1007/BF00116466

4. Besag J. Spatial Interaction and the Statistical Analysis of Lattice Systems. *J R Stat Soc Ser B Methodol*. 1974;36(2):192-236.

5. Leroux BG, Lei X, Breslow N. Estimation of Disease Rates in Small Areas: A new Mixed Model for Spatial Dependence. In: Halloran ME, Berry D, eds. *Statistical Models in Epidemiology, the Environment, and Clinical Trials*. The IMA Volumes in Mathematics and its Applications. Springer; 2000:179-191. doi:10.1007/978-1-4612-1284-3_4

6. Lindgren F, Rue H. Bayesian Spatial Modelling with R-INLA. *J Stat Softw*. 2015;63(1):1-25. doi:10.18637/jss.v063.i19

The summary statistics of individual SES by advantage index quartiles

| **Variable** | **First quartile (n=398)** | **Second quartile (n=375)** | **Third quartile (n=402)** | **Fourth quartile (n=378)** | **Overall (n=1568)** |
| --- | --- | --- | --- | --- | --- |
| **A1C_diff** |  |  |  |  |  |
| Mean (CV%) | 1.51 (141.3%) | 1.55 (134.6%) | 1.39 (155.3%) | 1.40 (144.8%) | 1.46 (143.5%) |
| Median [Q1, Q3] | 1.25 [0.200, 2.60] | 1.30 [0.200, 2.70] | 1.25 [0.00, 2.50] | 1.30 [0.200, 2.50] | 1.30 [0.100, 2.60] |
| **FirstA1Cv** |  |  |  |  |  |
| Mean (CV%) | 10.2 (16.0%) | 10.2 (15.4%) | 10.2 (16.8%) | 10.2 (15.9%) | 10.2 (16.0%) |
| Median [Q1, Q3] | 9.90 [8.90, 11.2] | 9.90 [9.00, 11.5] | 10.0 [8.80, 11.4] | 10.0 [8.80, 11.2] | 9.90 [8.90, 11.3] |
| **Age_category** |  |  |  |  |  |
| 60 or greater | 71 (17.8%) | 74 (19.7%) | 79 (19.7%) | 75 (19.8%) | 302 (19.3%) |
| Between 18 and 39 | 73 (18.3%) | 54 (14.4%) | 54 (13.4%) | 59 (15.6%) | 241 (15.4%) |
| Between 40 and 49 | 113 (28.4%) | 108 (28.8%) | 119 (29.6%) | 105 (27.8%) | 448 (28.6%) |
| Between 50 and 59 | 141 (35.4%) | 139 (37.1%) | 150 (37.3%) | 139 (36.8%) | 577 (36.8%) |
| **Gender** |  |  |  |  |  |
| Female | 272 (68.3%) | 235 (62.7%) | 272 (67.7%) | 245 (64.8%) | 1030 (65.7%) |
| Male | 126 (31.7%) | 140 (37.3%) | 130 (32.3%) | 133 (35.2%) | 538 (34.3%) |
| **Marital_status** |  |  |  |  |  |
| Divorced | 20 (5.0%) | 26 (6.9%) | 26 (6.5%) | 29 (7.7%) | 102 (6.5%) |
| Married | 248 (62.3%) | 221 (58.9%) | 242 (60.2%) | 230 (60.8%) | 953 (60.8%) |
| Separated/Widowed | 43 (10.8%) | 46 (12.3%) | 47 (11.7%) | 38 (10.1%) | 175 (11.2%) |
| Single | 78 (19.6%) | 76 (20.3%) | 78 (19.4%) | 78 (20.6%) | 311 (19.8%) |
| Missing | 9 (2.3%) | 6 (1.6%) | 9 (2.2%) | 3 (0.8%) | 27 (1.7%) |
| **Language** |  |  |  |  |  |
| English | 80 (20.1%) | 133 (35.5%) | 154 (38.3%) | 161 (42.6%) | 530 (33.8%) |
| Spanish | 318 (79.9%) | 242 (64.5%) | 248 (61.7%) | 217 (57.4%) | 1038 (66.2%) |
| **Education** |  |  |  |  |  |
| 8th grade or less | 188 (47.2%) | 148 (39.5%) | 160 (39.8%) | 134 (35.4%) | 638 (40.7%) |
| High school graduate/GED | 62 (15.6%) | 65 (17.3%) | 66 (16.4%) | 67 (17.7%) | 261 (16.6%) |
| Some college/College degree/Graduate degree | 32 (8.0%) | 63 (16.8%) | 73 (18.2%) | 71 (18.8%) | 241 (15.4%) |
| Some high school | 68 (17.1%) | 72 (19.2%) | 67 (16.7%) | 70 (18.5%) | 278 (17.7%) |
| Missing | 48 (12.1%) | 27 (7.2%) | 36 (9.0%) | 36 (9.5%) | 150 (9.6%) |
| **Employment** |  |  |  |  |  |
| Disabled/Retired | 26 (6.5%) | 34 (9.1%) | 35 (8.7%) | 33 (8.7%) | 129 (8.2%) |
| Employed | 94 (23.6%) | 102 (27.2%) | 106 (26.4%) | 106 (28.0%) | 415 (26.5%) |
| Other | 57 (14.3%) | 55 (14.7%) | 55 (13.7%) | 52 (13.8%) | 220 (14.0%) |
| Unemployed | 181 (45.5%) | 161 (42.9%) | 171 (42.5%) | 158 (41.8%) | 675 (43.0%) |
| Missing | 40 (10.1%) | 23 (6.1%) | 35 (8.7%) | 29 (7.7%) | 129 (8.2%) |
| **Housing** |  |  |  |  |  |
| Other | 36 (9.0%) | 41 (10.9%) | 38 (9.5%) | 30 (7.9%) | 146 (9.3%) |
| Own | 176 (44.2%) | 177 (47.2%) | 177 (44.0%) | 179 (47.4%) | 714 (45.5%) |
| Rent | 110 (27.6%) | 116 (30.9%) | 130 (32.3%) | 114 (30.2%) | 475 (30.3%) |
| Missing | 76 (19.1%) | 41 (10.9%) | 57 (14.2%) | 55 (14.6%) | 233 (14.9%) |
| **Insurance_status1** |  |  |  |  |  |
| No | 319 (80.2%) | 287 (76.5%) | 317 (78.9%) | 295 (78.0%) | 1228 (78.3%) |
| Yes | 58 (14.6%) | 75 (20.0%) | 70 (17.4%) | 66 (17.5%) | 273 (17.4%) |
| Missing | 21 (5.3%) | 13 (3.5%) | 15 (3.7%) | 17 (4.5%) | 67 (4.3%) |
| **DSME_class** |  |  |  |  |  |
| Mean (CV%) | 5.68 (78.0%) | 5.48 (73.1%) | 5.20 (84.0%) | 5.25 (86.0%) | 5.41 (79.9%) |
| Median [Q1, Q3] | 6.00 [3.00, 7.00] | 6.00 [2.50, 7.00] | 6.00 [2.00, 7.00] | 6.00 [2.00, 7.00] | 6.00 [2.00, 7.00] |
| **stay_days** |  |  |  |  |  |
| Mean (CV%) | 409 (34.1%) | 406 (32.5%) | 411 (32.4%) | 397 (33.1%) | 406 (33.0%) |
| Median [Q1, Q3] | 392 [358, 458] | 384 [357, 462] | 397 [357, 465] | 384 [357, 462] | 387 [357, 461] |
| Missing | 2 (0.5%) | 8 (2.1%) | 2 (0.5%) | 3 (0.8%) | 15 (1.0%) |
| **Lack_of_transportation** |  |  |  |  |  |
| No | 349 (87.7%) | 338 (90.1%) | 362 (90.0%) | 344 (91.0%) | 1406 (89.7%) |
| Yes | 39 (9.8%) | 28 (7.5%) | 32 (8.0%) | 27 (7.1%) | 128 (8.2%) |
| Missing | 10 (2.5%) | 9 (2.4%) | 8 (2.0%) | 7 (1.9%) | 34 (2.2%) |
| **Lack_of_social_support** |  |  |  |  |  |
| No | 313 (78.6%) | 306 (81.6%) | 342 (85.1%) | 318 (84.1%) | 1291 (82.3%) |
| Yes | 75 (18.8%) | 60 (16.0%) | 52 (12.9%) | 53 (14.0%) | 243 (15.5%) |
| Missing | 10 (2.5%) | 9 (2.4%) | 8 (2.0%) | 7 (1.9%) | 34 (2.2%) |
| **Coronary_heart_disease** |  |  |  |  |  |
| No | 369 (92.7%) | 335 (89.3%) | 364 (90.5%) | 346 (91.5%) | 1428 (91.1%) |
| Yes | 16 (4.0%) | 32 (8.5%) | 23 (5.7%) | 21 (5.6%) | 93 (5.9%) |
| Missing | 13 (3.3%) | 8 (2.1%) | 15 (3.7%) | 11 (2.9%) | 47 (3.0%) |
| **High_blood_pressure** |  |  |  |  |  |
| No | 195 (49.0%) | 157 (41.9%) | 166 (41.3%) | 178 (47.1%) | 701 (44.7%) |
| Yes | 190 (47.7%) | 207 (55.2%) | 222 (55.2%) | 189 (50.0%) | 817 (52.1%) |
| Missing | 13 (3.3%) | 11 (2.9%) | 14 (3.5%) | 11 (2.9%) | 50 (3.2%) |

|  |  |
| --- | --- |
| Variable | Multilevel BYM with interaction |
| Baseline hba1c | 0.699 (0.647, 0.751) |
| Duration (<439 days) | 0.004 (0.003, 0.005) |
| Duration (>=439 days) | 0.001 (0, 0.001) |
| Language |  |
| English | reference |
| Spanish | -0.159 (-0.378, 0.059) |
| Age |  |
| 60 and greater | Reference |
| Between 18 and 39 | -0.591 (-0.895, -0.288) |
| Between 40 and 49 | -0.446 (-0.703, -0.189) |
| Between 50 and 59 | -0.325 (-0.567, -0.084) |
| Marital status |  |
| Married | reference |
| Divorced | -0.297 (-0.646, 0.051) |
| Separated/ widowed | -0.44 (-0.721, -0.16) |
| Single | -0.19 (-0.411, 0.032) |
| Education |  |
| 8^th^ grade or less | reference |
| High school graduate | 0.094 (-0.403, 0.59) |
| College/ graduate degree | -0.194 (-0.77, 0.382) |
| Some high school | -0.052 (-0.522, 0.418) |
| DSME class | 0.031 (0.011, 0.051) |
| Advantage rank | -0.019 (-0.03, -0.009) |
| Mica rank | 0.018 (0.007, 0.03) |
| Mobility rank | 0.003 (0, 0.007) |
| Opportunity rank | 0.005 (0.001, 0.009) |
| Education*Advantage Rank |  |
| 8^th^ grade or less | REFERENCE |
| High school graduate | 0.001 (-0.008, 0.01) |
| College/ graduate degree | 0.006 (-0.003, 0.016) |
| Some high school | 0.005 (-0.003, 0.014) |
